# Supplementary material for: Neural dynamics differentially encode phrases and sentences during spoken language comprehension
Source: PLoS Biol. 2022 Jul 14;20(7):e3001713. doi: 10.1371/journal.pbio.3001713 (PMC9282610; doi:10.1371/journal.pbio.3001713)
Supplement: S1 Appendix — (PDF) [file pbio.3001713.s001.pdf]

Appendix 1: All the phrase-sentence pairs that were used in the experiment

1, de blauwe bal (the blue ball) / de bal is blauw (the ball is blue)

2, de blauwe knoop (the blue button) / de knoop is blauw (the button is blue)

3, de blauwe sok (the blue sock) / de sok is blauw (the sock is blue)

4, de blauwe strik (the blue bow) / de strik is blauw (the bow is blue)

5, de blauwe stoel (the blue chair) / de stoel is blauw (the chair is blue)

6, de blauwe pijl (the blue arrow) / de pijl is blauw (the arrow is blue)

7, de blauwe bel (the blue bell) / de bel is blauw (the bell is blue)

8, de blauwe helm (the blue helmet) / de helm is blauw (the helmet is blue)

9, de blauwe boot (the blue boat) / de boot is blauw (the boat is blue)

10, de blauwe mok (the blue mug) / de mok is blauw (the mug is blue)

11, de groene tas (the green purse) / de tas is groen (the purse is green)

12, de groene laars (the green boot) / de laars is groen (the boot is green)

13, de groene bijl (the green ax) / de bijl is groen (the ax is green)

14, de groene schoen (the green shoe) / de schoen is groen (the shoe is green)

15, de groene ster (the green star) / de ster is groen (the star is green)

16, de groene kom (the green bowl) / de kom is groen (the bowl is green)

17, de groene deur (the green door) / de deur is groen (the door is green)

18, de groene kam (the green comb) / de kam is groen (the comb is green)

19, de groene pen (the green pen) / de pen is groen (the pen is green)

20, de groene brug (the green bridge) / de brug is groen (the bridge is green)

21, de rode trui (the red sweater) / de trui is rood (the sweater is red)

22, de rode doos (the red box) / de doos is rood (the box is red)

23, de rode vaas (the red vase) / de vaas is rood (the vase is red)

24, de rode vis (the red fish) / de vis is rood (the fish is red)

25, de rode bank (the red couch) / de bank is rood (the couch is red)

26, de rode mier (the red ant) / de mier is rood (the ant is red)

27, de rode bus (the red bus) / de bus is rood (the bus is red)

28, de rode tent (the red tent) / de tent is rood (the tent is red)

29, de rode bloem (the red flower) / de bloem is rood (the flower is red)

30, de rode draak (the red dragon) / de draak is rood (the dragon is red)

31, de gele hoed (the yellow hat) / de hoed is geel (the hat is yellow)

32, de gele riem (the yellow belt) / de riem is geel (the belt is yellow)

33, de gele sjaal (the yellow scarf) / de sjaal is geel (the scarf is yellow)

34, de gele broek (the yellow pants) / de broek is geel (the pants is yellow)

35, de gele kan (the yellow pitcher) / de kan is geel (the pitcher is yellow)

36, de gele lamp (the yellow lamp) / de lamp is geel (the lamp is yellow)

37, de gele eend (the yellow duck) / de eend is geel (the duck is yellow)

38, de gele jas (the yellow jacket) / de jas is geel (the jacket is yellow)

39, de gele fles (the yellow bottle) / de fles is geel (the bottle is yellow)

40, de gele fiets (the yellow bicycle) / de fiets is geel (the bicycle is yellow)

41, de paarse vlag (the purple flag) / de vlag is paars (the flag is purple)

42, de paarse tas (the purple bag) / de tas is paars (the bag is purple)

43, de paarse mand (the purple basket) / de mand is paars (the basket is purple)

44, de paarse jurk (the purple dress) / de jurk is paars (the dress is purple)

45, de paarse bril (the purple glasses) / de bril is paars (the glasses is purple)

46, de paarse kerk (the purple church) / de kerk is paars (the church is purple)

47, de paarse pop (the purple doll) / de pop is paars (the doll is purple)

48, de paarse muur (the purple wall) / de muur is paars (the wall is purple)

49, de paarse veer (the purple feather) / de veer is paars (the feather is purple)

50, de paarse kast (the purple closet) / de kast is paars (the closet is purple)
