# Supplementary material for: Neural dynamics differentially encode phrases and sentences during spoken language comprehension
Source: PLoS Biol. 2022 Jul 14;20(7):e3001713. doi: 10.1371/journal.pbio.3001713 (PMC9282610; doi:10.1371/journal.pbio.3001713)
Supplement: Attachment — Submitted filename: Response_to_Reviewers_2022_0402.pdf [file pbio.3001713.s002.pdf]

[PBIOLOGY-D-21-01699R1]
Reviewer #1:

The authors set out to understand whether complete sentence structures (e.g., The vase is red) and minimal phrases (e.g., The red vase) elicit distinct low frequency oscillations in neural responses. They investigate this question in terms of phase coherence within trials, between brain areas, between lower and higher frequencies and in terms of power in the alpha band. They also investigated the encoding of acoustic detail across the two conditions. The authors find that phase coherence is greater across sentence trials as compared to phrase trials that the right posterior sensors were differentially coherent in phrase and sentence conditions, and alpha was more inhibited in the phrase condition. Furthermore, different delays in the spectral-temporal receptive field model distinguished phrase and sentence conditions across subjects. Overall the authors conclude that phrases and sentences elicit different responses in the power and phase of frequency-based metrics of neural activity.

The work is well-conducted and the analyses are sophisticated. My main hesitation relates to the motivation of the analyses presented here, and the corresponding interpretation of the results.

MAJOR

(1) Linking hypothesis

(Q1)*My main concern is that the motivation of the analyses conducted and the neural metrics used is not clear. Consequently, the interpretation of the results is also not clear.*

(A1) We thank the reviewer for these clarification points; we indeed make directed hypotheses on pages 5-12 of the Introduction and have revised this section, and the Abstract, for clarity. Our experiment tests the predictions of a computational model published in this journal (Martin & Doumas, 2017), which proposed a mechanism for building syntactic structure, time-based binding. The model makes the prediction that the processing of hierarchical syntactic units increases phase synchronization as a function of number of constituents. The model's architecture also predicts that connectivity would increase because information flows between related syntactic nodes to across time, which in turns represent a constituent. We regret that that was not clear in the first submission of the manuscript and have strengthened our outline of the

predictions in the manuscript on pages 5-13. In sum, existing theoretical and computational models (viz., Martin & Doumas, 2017, Martin 2016, 2020) predicted that these differences should principally occur in measures of phase synchronization and connectivity, which we observed.

(Q2)*What is the underlying mechanism that would lead to differences in phase coherence across phrases and sentences, for example? It might be helpful for the authors to outline a set of predictions in the introduction, to motivate the experimental manipulations and link the different outcomes directly to different hypotheses.*

(A2) Thank you for raising this interesting point. Many studies in the literature have demonstrated the importance of phase modulation in sentence comprehension (e.g., Brennan & Martin, 2020; Giraud & Poeppel, 2012; Howard & Poeppel, 2012; Meyer et al. 2017; Kaufeld et al., 2020; Keitel et al. 2018, 2020; Luo & Poeppel, 2007; Molinaro et al. 2013; Peelle & Davis, 2012).

Our goal is to work towards identifying a mechanism, however, we note that most research on neural oscillations presents empirical phenomena without a proposed mechanism. In the neural oscillations literature, it is still an open question whether different syntactic structure would induce measurable differences in neural responses. To our knowledge, this has not been shown to date and is crucial under the hypothesis that neural oscillations reflect syntactic structure building.

Our main hypothesis (derived from Martin & Doumas, 2017 and Martin, 2020) is derived from a mechanism. The hypothesis that additional constituents and hierarchical relations in sentences compared to phrases should modulate power, phase, and connectivity comes about because of how information is passed in a neural network that groups representations together in time; our results reported here are thus consistent with the time-based binding mechanism proposed by Martin & Doumas (2017) and Martin (2020). However, more empirical evidence is necessary before a single mechanism can be unequivocally supported and others excluded.

Ding et.al. 2016, left the potential mechanism of the phenomenon as an open question and as such, we see this manuscript as laying important first groundwork towards understanding how neural dynamics come to reflect syntactic structure within an existing mechanistic theoretical framework.

The reason/motivation for presenting different metrics of neural dynamics is that low frequency oscillations are quite well-observed in previous research to reflect spoken language processing. However, whether they reflect physically “subtle” but structurally substantial differences in syntax, has not been tested. Previous research has demonstrated sensitivity to the presence and absence of syntactic structure, but not to the nuances of it. Taking a comprehensive approach to analyzing neural readout allowed us to show that phase synchronization and power connectivity differed parametrically between phrases and sentences.

We have revised the Introduction (see pages 5-13) and Abstract to clarify our hypothesis, including its relation to mechanism, per the reviewer’s suggestion.

(2) Acoustic differences across conditions and within conditions

I have some concerns regarding the acoustic "matching" across conditions. ***(Q1)*** *Were phrases and sentences normalised to be the same physical length?*

(A1) First, we did indeed match the physical length across different types of stimulus; we have clarified this further in the Methods section. However, unfortunately there is no existing method can normalize the physical length of every stimuli to be exactly the same without changing the acoustic properties, e.g., the sampling rate. What we did is artificially synthesized the speech stimuli with the same sampling rate then truncate or zero-pad them to 1-second to make sure the processing duration would be identical. Our normalization method has also been used in many impactful studies, e.g., Ding et al (2016) (Gui et al., 2020 *Nat. Neuro*; Jin et al., 2020 *eLife*; Jin et al. 2018 *Nat. Comms.* In Ding et al., they also normalized each syllable to be 250 ms in duration with the same manipulations. For checking if our 1 Hz manipulation is effective, we have conducted a frequency tagging analysis as now shown in Fig 1k and Fig 1l, the comparison indicates that the 1-second normalization is effective. Please see the new Figure 1.

(Q2) *Was prosody and stress accounted for?*

(A2) Yes. To properly demonstrate that acoustic confounds are eliminated, more so than in any related existing empirical work, we have conducted an additional time-wise similarity analysis for all the stimuli in both temporal and spectral dimension, as now shown in Fig 1. Please note that this analysis demonstrates the similarity of our stimuli above and beyond what has been

used as a benchmark in the literature (as published in this journal Keitel & Gross, 2018, Keitel, Gross, & Kayser, 2018), and Ding et al. 2016).
The highly similar pattern in temporal dimension (cosine similarity always greater than 0.9 in each sounding time point, minimally; note that the y-axis begins at 0.9 and plots the cosine similarity to 1.0, approaching identity) between the phrases and sentences shows that the phrase-sentence pairs were controlled to be as physically similar as possible while still being unequivocally phrases and sentences. This temporal analysis demonstrates that lexical and/or prosodic stress, and momentary intensity, cannot explain the differences we observe in the neural signal. We have added this analysis to the manuscript in Figure 1.

The spectrum of temporal envelope reflects both prosodic information and acoustic modulations. Ding et al (2016),demonstrated that they had effectively had prosody-neutral synthesized speech by calculating the modulation spectrum of speech stimulus and presenting that. They showed that their stimuli only features a 4-Hz peak in intensity/power of the stimuli (i.e., corresponding to the presentation rate of the syllables), whereas an additional peak at 2 Hz (i.e., the presentation rate of words) in neural responses, made their conclusion meaningful (because physical demarcation of the words' rate was not present in the stimuli).

In the current study, in order to demonstrate that prosody neither creates a significant physical difference in our stimuli nor can account for the modulations of neural readout that we report, we have added a frequency decomposition on the temporal envelope for both condition, as shown in Fig 1h. Statistical analysis failed to show any significant differences across conditions in every single frequency; above and beyond existing benchmarks, we also show that this is true of our stimuli across time.

In addition, we now include a Bayesian inference statistical analysis where we show that phrases and sentences are indistinguishable acoustically. We have added this analysis to the manuscript and describe it on pages 5-8, 31-36, the revised Methods section, and in Appendix 2. By taking a Bayesian inference approach, we can demonstrate that there is evidence for the null hypothesis that the conditions are acoustically indistinguishable from a distributional point of view .

Given the acoustic similarity and lack of statistical differences in our stimuli, it is difficult to see how our pattern of effects in the neural readout could be attributed to prosody or stress patterns that were not measurably present in

the stimulus. This is because prosodic modulations and stress patterns are physical properties of the stimuli. Furthermore, if there were indeed an effect reflecting these physical features, it should be observed bi-laterally (Cogan et al., 2014; Hickok & Poeppel, 2007, 2016; Peelle, 2012). For instance, in Ding et.al (2016), the frequency tagging to syllables or backward-played syllables was observed when participants listened to their unknown language, which was bilaterally distributed without hemispherical dominance. In addition, to capture the neural characteristics of prosody, stimuli must be longer in time, e.g., 7 seconds with seven 1-hz cycles. It is difficult to see how such a prosodic effect could be reflected in our 1-second stimuli.

***(Q3)** Were the shared words across conditions identical acoustic tokens, or pronounced differently?*
(A3) They were identical as they were produced by a speech synthesizer.

(Q3 con't) It might be useful to conduct an acoustic analysis on the trials across conditions to assess how well matched they actually are. One concern is that a difference in lexical stress — i.e. momentary intensity, even though the sentence in its entirety is matched — could explain increased phase coherence across conditions, as well as the STRF result. This is potentially visible in Figure 9. It is possible that any kind of acoustic confound could explain the present results.

(A3) We thank the Reviewers for encouraging us to exhaustively demonstrate the physical similarity of our stimuli; we now present a set of new analyses of our stimuli which further demonstrate their maximal physical similarity (see Figure 1, the revised Methods, Appendix 2). For the suggestion of adding an analysis of the acoustic features comparison within condition and across conditions, we conducted a Representational Similarity Analysis (RSA), in which we compared not only the physical properties of phrase-sentence pairs by condition, we also compared all possible pairs in our stimuli across items, (Fig 1j). Using cosine similarity, which considers the matching of each dimension (time points in our case) in the vector space, our analysis indicates that the stimuli were highly similar in physical properties. We had now added these new analyses and figures to the manuscript in the form or Figure 1 and Appendix 2.

We would also like to add that our STRF analysis explicitly models the speech envelope as a predictor as neural response. , if there were acoustic differences (our new analysis now demonstrates that there are not), they

would have been taken into account and modeled in the same sense that regression models do (see Appendix 2).

Phase-related activity is interpreted as a reflection of the temporal consistency of the firing of neurons (e.g., McLelland & Paulsen, 2009; Ng, Logothetis, & Kayser, 2013). Phase coherence could be driven by any phase-locked process, either by a physical property (N1 or M100) of the stimuli, or by higher-level linguistic processing of the stimuli at a fixed time point (e.g., in the classical psycholinguistic literature, cue-based grammatical or predictive anomalies as reflected in the N400, or syntactic integration and/or revision of structural relationships as reflected in the P600). In fact, observing modulations of phase that are either consistent or inconsistent with that of the stimuli is not necessary to induce neural synchronization; differences in phase coherence can be induced using the same physical stimuli (4-hz), which introduce different patterns of 1-hz phase coherence (Jin et al., 2018).

Relatedly, ***(Q4)*** *how similar were the trials within a condition? If they were quite heterogeneous, either acoustically or in terms of when linguistic events occur, I am not sure why you would expect phase -i.e. the timing of the syntactic process of interest- to be the same across trials.*

(A4) The linguistic events of units of each conditions always occurred at the same time, as the speech stimulus was synthesized and controlled in order to assure maximal uniformity despite different lexical content by item and the fact that we did not force isochrony on the stimuli. The RSA analysis in Figure 1g compared all possible pairs (viz., phrases to phrases, phrases to sentences, and sentences to sentences). There is no significant difference in the distributional properties of the physical characteristics of the stimuli either between conditions or within them. Please see Figure 1 and Appendix 2 for details of the set of analyses.

(3) Framing of oscillations

Neural oscillations of course play a big role in this paper. There were a few times where I felt like different "types" of oscillations were being put into the same bundle, or at least were not fully motivated. For example, the low frequencies investigated in Ding et al., 2016 arose through entrainment: the stimulus was manipulated such that any putative syntactic operation would occur rhythmically at the experimental-determined rate. If the stimulus was

sped up or slowed down, the corresponding computation would also happen faster or slower in direct correspondence. ***(Q1)*** *Here, the stimulus was not manipulated in its rhythmicity, and so the same entrainment process would not be expected to occur. Instead, if I am understanding correctly, the authors expect that certain neural operations "naturally reside" within certain frequency bands of the neural signal. In the same way that responses in the alpha band have been linked to attention, perhaps responses in lower frequencies can be linked to syntactic operations, or the emergence of higher order structure.*

(A1) The reviewer indeed states the argument we would like to make from our results. Some models (Martin & Doumas, 2017; Martin, 2020) try to link low frequency oscillations to the building or emergence of structure. Existing studies in the oscillations literature on sentence processing (see Meyer, 2018 for a review) also tie modulations of delta power to syntactic structure, but those studies did not a) control the physical or semantic properties of the stimulus, b) parametrically vary syntactic structure, or c) check other dimensions of the neural readout besides power (specifically, phase and connectivity) to see if manipulating the number and type of syntactic constituents systematically affected them.

The core finding of Ding et al. (2016) is that the building of syntactic structure is a function of its rate of occurrence. However, the paper did not talk about what would happen if two syntactically *different structures appear at the same rate* (in our case, at 1Hz). Our hypothesis was that syntactic structures should be reflected in phase and connectivity dimensions of the neural response, even if the physical properties are indistinguishable (and thus would appear identical in a frequency tagging analysis). Our results demonstrated that using this popular approach, known as frequency tagging, can make it quite difficult to detect the processing differences between syntactic structures when they occur at the same rate - which happens often in natural speech since words, phrases, and sentences often have similar timescales.

(Q2) *I think this is another area where the authors could be more concrete about why certain signals were chosen to be analysed, and crucially what neural process that signal putatively reflects.*

(A2) Thank you for pushing us to be more concrete. Low frequency neural responses (viz., the delta band) are typically analysed in the literature on spoken language processing. But there is no agreement or consensus in the

literature as to what delta band modulations, either in phase or power, indicate. It is also not clear whether there is a 1-to-1 relationship between a modulation in a given neural readout and a neural or computational process. Our work entered new territory by letting the data guiding us to find the readouts of interest, under the broad predictions of a computational model, , as the existing literature does not give a clear starting point of where to look for effects of syntactic structure other than on the theta-delta timescale. For orthogonality, our regions of interest (ROI) were all selected independent of conditions. All parametric statistical analysis were based on grand average of the data, all non-parametric statistical analysis were conducted in a data guiding approach. To eliminate the potential double-dipping risks, we have shown how the sensors were selected, e.g., Fig 4b, Fig 6c, etc. We also listed the references which described what the activity in our selected ROI might reflect, and what we are new that found in our study.

MINOR

it would be useful to see a full list of stimuli

Thank you for pointing this out. We have added all the stimuli that used in this study, please see Appendix 1.

Reviewer #2, William Matchin: SUMMARY

The authors report an EEG experiment aimed at testing "whether low frequency neural oscillations reflect differences in syntactic structure". They compared two stimulus types: phrases and sentences, that were roughly matched for conceptual content, equal in overall energy, but differed in syntactic structure. Previous research assessing a similar question, namely the widely-cited study of Ding et al. (2016), looked at low frequency power, finding peaks in the power spectrum corresponding to the intelligible linguistic structure of the stimulus, going beyond the perceptual (i.e. syllabic) frequency of the stimulus. This study examined a large number of dimensions beyond spectral power (e.g., phase coherence, connectivity, phase-amplitude coupling, etc.) and ascertained whether these dimensions distinguished between the stimulus types, presumably due to some aspect of syntactic encoding. They in fact found a variety of significant effects which seemingly correspond in some way to syntactic processing, and some effects which did not (e.g. phase-amplitude coupling), and some effects which were diminished

in sentences (alpha band power).

EVALUATION

This study is a nice exploratory look into the electrophysiological measures which reflect syntactic processing. I think that the statements in the summary section accurately represent this manuscript's contribution: the differentiation between different degrees of syntactic structure is reflected in multiple dimensions of brain activity. The authors are appropriately tentative in their interpretations of the results, not taking them necessarily as mechanisms of syntactic computation but as readouts of those computations. This is a fairly comprehensive look at such readouts, and the authors are to be commended for the methodological rigor of their study. The analyses seem to be rigorously performed, although I am not an expert in electrophysiological data analysis and would hope that another reviewer who is more of an expert in electrophysiology can assess these methodological details. I do have some concerns, detailed below, that the authors should address in a revision.

1. Most importantly, the stimuli are not perfectly controlled for lexical differences. While the stimuli are decently controlled at the acoustic and phonological levels, there is an additional terminal node in the sentence condition ("is") that is absent from the phrase condition. Ideally these conditions would be perfectly matched on the lexical level, or additional data collected that address this concern. How much of the results are due to simply additional lexical processing in the sentence condition?

We thank the Reviewer for making this point. It would indeed be ideal if it were possible for there not to be the additional word ‘is’ between the phrase and sentence conditions. Unfortunately, we do not believe that is possible (at least in Dutch) to perfectly match lexical content (which we have done except for ‘is’) while also forming a phrase and sentence that are closely matched semantically and physically and are the same length in time (the factors which create the condition manipulation). In fact, the addition of the terminal node is the condition manipulation we induced in order to test for an effect on neural readout as a function of number and type of constituents.

We understand the Reviewer’s concern that our effects might be driven by the addition of ‘is’. But in the sense that our effects are driven by our condition manipulation, the addition of ‘is’ is also our condition manipulation. Other than the word ‘is’, our stimuli are matched in word frequency because they contain the same words.

While we cannot completely rule out that our effects are driven by the presence of ‘is’ as a word but not as part of the sentence, nor the role of the inflectional morpheme /e/ in the phrase condition, there are two arguments against the interpretation that our affects are driven only by the lexical nature of ‘is’ and not by the fact that ‘is’ reflects the sentence and its structure building. First, we observe differences between phrase and sentence conditions before the onset of the word ‘is’ (in phase coherence), as well as after (in both phase coherence and power connectivity). This likely reflects differences in structure and meaning that come from building a phrase versus a sentence, and suggests that differences are not solely dependent on the onset or encountering of ‘is’.

Secondly, in our new acoustic analyses, we can show that there was not a single time point in the physical stimulus that differed between these two types of stimuli, including when the word ‘is’ occurred. To our knowledge, there is no study in the psycholinguistic literature that has shown that people prefer to interpret a lexical item by itself in the context of the sentence. In fact, sentence processing is regarded as an automatic process such that words are automatically integrated during sentence comprehension (Bonhage et al., 2017; Pulvermüller & Shtyrov, 2003; Shtyrov et al., 2012).

2. it would seem important to me to include an unstructured control stimulus, e.g. a scrambled sentence or other word list that controls for lexical properties to determine if the phase increases actually reflect syntactic structure - a straightforward prediction would be that contrasting the phrase condition and a less structured list condition should result in the same kind of phase coherence effects as that observed here. In fact, such a contrast would probably better control for lexical confounds given that the exact same set of lexical stimuli could be used.

We thank the reviewer for raising this important point. We had tested sentences vs. word lists with the exact same lexical items (see Kaufeld et al. 2020), and found differences in phase mutual information, suggesting that phase synchronization is relevant for sentence processing compared to word list processing. However, it is not possible to compare constituent number and type between sentences and word lists, as word lists do not have any syntactic structure. We note two things: first, our stimuli do contain the same words except for ‘is’, and second, a word list condition in our design here would give us the difference between processing a grammatical structure (phrase, sentence) and an ungrammatical one without any syntax (word list).

We feel this difference between stimuli is too great to know what to attribute any differences in neural response to (for example, any difference could be due to processing a grammatical sentence which entails processing more than syntax and allows for more predictive processing than a word list, or the lack of syntactic structure altogether, or participants memorizing word pairs or triplets, or participants trying to repair word lists into sentences, or all four interpretations across different groups of participants).

As such, a word list condition seems to complicate the interpretation of the neural response more than directly addressing our question about differences in neural response to grammatical syntactic structures that differ in number and type of constituents. In sum, by adding a word list condition, one could measure the difference between the processing a grammatical structure and the processing an ungrammatical one, but not the difference between syntactic structures we were after.

Furthermore, in natural speech processing, prediction likely plays an important role; prediction cannot occur in a ‘random word list’ as it does in a sentence or phrase because a wordlist lacks the syntactic and semantic structure on which to base predictions; this is another crucial difference that would be introduced by a word list condition. Additionally, for stimuli with a small number of words, e.g., 3 or 4 as in our stimuli, adding a random word list condition would introduce the possibility that participants repair or recover a grammatical structure akin to the sentence or phrase condition. For instance, if the list is ‘ball blue the’, one might recover it to ‘the blue ball’, which would introduce more uncertainty into interpretation of the neural response.

Instead of adding this ‘random word list’ condition, what we should show, as the reviewer suggested, is the similar acoustic/physical property of the two types of stimuli. For this purposes, we have compared the stimuli in both time and frequency domain with various measures to make sure that our effect is definitely not driven by acoustic differences (see Figure 1 and Appendix 2). We hope the new analyses successfully assuage the reviewers’ concerns and are seen as clear evidence that the effects we found reflect syntactic structure differences between phrases and sentences.

3. Both the limited stimulus set (five color adjectives) and metalinguistic task (including a phrase vs. sentence judgment) raise some potential concerns about formulaic processing. Did subjects adopt a strategy whereby they looked for an "is" in the sentence condition, for example?

The reason we used three types of task is that we wanted to prevent participants paying attention to only one of the key features in the stimuli. Because the participants do not know in advance which task they will be asked to perform, they cannot adopt a task-specific strategy when listening to the speech stimulus. The strategy of only looking for 'is' is not helpful for task types 2 and 3 (color and object tasks), which outnumber the linguistic task 2 to 1. Our three types of task were evenly and randomly assigned across each blocks. Furthermore, our behavioral result do not support this hypothesis. The overall accuracy and RT showed the same pattern across conditions, indicating a uniformity in performance across tasks.

4. There is insufficient detail about the stimuli in the study. Did the entire stimulus set consist of stimuli like the following: the-ADJ-NOUN and the-NOUN-is-ADJ, or were other structures used?

Our apologies that this was not sufficiently clear. These were the only structures used and this is now further emphasized in the Methods section. The complete list of stimuli is now available in Appendix I.

What is the nature of gender agreement in Dutch - is this present in both the phrase and sentence conditions? This should be explained and illustrated in greater detail, including a full gloss.

We have now added an Appendix I which lists all the stimuli that were used in the experiment. Dutch gender agreement, which is present in the phrase condition, is what allows stimuli to be matched in time and number of syllables. Without it, comparison of differing syntactic structure in the same amount of time and syllables would not be possible. Both conditions contain agreement, however, with sentences requiring person and number agreement and phrases requiring gender and number agreement. We have added a gloss to the syllable level in Figure 1.

In order to test for physical differences stemming from agreement and verb morphology (/e/ vs. /is/), we compared the acoustic features between conditions in both temporal and spectral dimension (see Figure 1). In addition to comparing the phrase-sentences pairs as we mentioned earlier in this response, we also performed a full-cross comparison between all stimuli with computational simulations are now shown in Fig 1 and detailed in Appendix 2. All the comparisons are point to one direction, which is our stimuli are indistinguishable from each other, both within and across conditions, in

physical properties. This suggests that any difference we find in the neural response is primarily driven by differences in cognitive transformations related to language processing.

5. In discussion of the phase connectivity results, the authors discuss a very late right-lateralized response. The same with the TRF results. It is highly doubtful to me that this late right-lateralized response reflects syntactic processing per se, given that other methods (hemodynamic, aphasiology) strongly associated left hemisphere regions with syntax, and the lateness of the effect (well after the sentence ends) suggest a post-stimulus processing effect of some kind.

We thank the reviewer for raising this interesting point for discussion. We agree and hesitate to infer computational process based on temporal or regional effects alone. First, the phase and power connectivity effects are observed in two waves, the first before the end of the sentence, and the second after. It is very plausible that these two time windows of effects reflect different underlying processes, but more work closely comparing syntactic structure while controlling for physical properties and semantic properties is needed to equivocally rule out these competing interpretations. As such, we have modified the discussion of the localization pattern in the Discussion, please see pages 50-51. We no longer imply that the later effects reflect unequivocally syntactic processing. We agree that a much more nuanced discussion of the effects is warranted.

5. Typo in Fig. 7i - Response in the title is spelled incorrectly.

Thank you for catching this, we have corrected it.

-William Matchin

Reviewer #3: *The manuscript "Neural encoding of phrases and sentences in spoken language comprehension" the authors describe an EEG experiment comparing several measures of brain activity between phrases and sentences. In my understanding, the unique feature of the paper is that the stimuli were closely matched acoustically (number of syllables, length) and on semantic content. In fact, there were two options: 'de rode vas' or 'de vas is rod', different stimuli were created by exchanging the object (vas) and the*

color (rod) but preserving the exact same structure.
I am not recommending the manuscript for publication, since I did not really understand the question and what the findings mean. This might be because I'm not an expert in syntax, but since the journal has a broader focus, I think it's a valid concern. I would like to explain in more detail what I didn't understand, and hope this is useful to the authors.
I did not understand, what question is addressed by the comparison between the two structures.

The main question we address is which neural readouts (or measurements of the neural response) are relevant for tracking the transformation of a physically near-identical (viz., statistically indistinguishable) stimuli into two different abstract structures. This process is interesting because it is an instance of brain computation where the brain takes a physical stimulus that would be identical to another species or machine, and computes different structural properties based on culturally-acquired abstract knowledge stored in the brain. In order to understand this neural computation better, an important first step (in addition to theoretical and computational modelling) is to know which neural readouts are relevant, in order to be able use those readouts in the future to constrain how we build our theories and models. We have added this explanation of the goal and importance of our work to the Introduction on page 5.

What I gather is, that the theoretical model (Martin and Doumas) is using a timing/oscillatory code to represent structural relations between constituents. First of all, it would be nice to mention what a constituent is, e.g. based on the example trees in Fig. 1. If by constituents you mean each word, or leafs of the tree, then there's three words on the left and four words on the right. If you mean every node then there's six on the left and eight on the right. Since it's mentioned that the number of constituents depends on the way the sentence/phrase is analyzed, you probably mean the nodes. For the comparison here it doesn't make a difference, because the right sentence has more words and more nodes. I don't know whether those two factors (number of words, number of nodes in the tree) could be even controlled/orthogonalized experimentally?

Thank you for raising this point and helping us clarify our work. The reviewer is correct, the number and types of nodes, and their relations, is what we manipulated. The reviewer correctly surmised that the number of words cannot be controlled to be the same and have the condition manipulation be

intact. But, the number of syllables (thought to the be primary unit in speech comprehension) and the amount of time the stimulus takes can be.

The recognition of words by the brain already represents the departure from physical stimulus properties to abstract brain computation, because many different physical instantiations can signify the same word (e.g., speakers of different gender identity and vocal tract length produce acoustic distinct tokens, but we recognize the same word regardless. This is a powerful form of filtering by the brain. Furthermore, the same speaker can produce acoustically distinct tokens simply as a function of production context). Thus, controlling for the number of syllables and acoustic properties was the most crucial starting point for us to be able to measure the neural readouts related to abstract structure computation in the brain.

But the hypothesis based on the model would be that the number of constituents (or words) you have to represent at a given time would be reflected in the power and synchronization of oscillations (page 5, line 106). I assume that means: more constituents more power/connectivity (that's not explicitly stated)?

Yes, this is the directional prediction. We have revised and emphasized our directional predictions further in the hypothesis section across pages 5-8 and in our specific research questions on pages 11 and 12.

Since there are only two example structures compared here, it's not only the number of constituents or words that's different. Also one of them is a sentence (has a verb), one of them a phrase; one of them has the object information first, the other one has the color information first.

Our stimuli are controlled as much as they can be in physical, temporal, and semantic dimensions while still allowing the formation of the condition manipulation that is crucial for our research question. We have now included new analyses of the physical and temporal properties of our stimuli and two statistical approaches to demonstrate that our stimuli are statistically indistinguishable in terms of their physical and temporal properties. This high degree of similarity in the physical and temporal domains is what allowed us to observe differences in neural readout that are not driven by physical or temporal differences, and by inference are likely to be related to the computation of the phrase and sentence meanings by the brain. To our knowledge, there has not been a demonstration that parameters of syntactic

structure (here, number and type of constituents) affect neural readout, partly because no study has created and used stimuli that were controlled in the physical and temporal domains outside of frequency tagging paradigms, which restrict results only to the power domain.

This is maybe why the authors don't make a directed hypothesis in most places of the manuscript. For example
"whether low frequency neural oscillations reflect differences in syntactic structure" (line 153)
"hypothesis that low frequency neural oscillations would be sensitive to the difference in syntactic structure of the phrases and sentences" (line 164)
So that means the question is: Is there a difference in brain activity between two specific syntactic structures, and what exactly the difference is; whether it is phase synchronization, PAC, amplitude correlation. That is of course a valid question, but I don't understand how one can link back to the model if there is no directed hypothesis. And if the hypothesis is, (line 106) that more constituents produce more power/synchronization, the comparison here doesn't test it. There are just two instances of structures, and they differ in the number of constituents, but also whether object and color information comes first, and whether it is a sentence (contains a verb) or a phrase.

We thank the reviewer for appreciating the validity of our research question. This is exactly the directed hypothesis which we test in our analyses; we found that theta band phase coherence discriminated between syntactic structures and that low frequency phase synchronization reflect functional power connectivity differences between phrases and sentences.
These results were previously unknown and give us new benchmark targets that models of sentence processing should explain.

Thank you for raising the point about more clarity for the syntactic tree in Figure 1. We have now included the syllable level which most closely corresponds to the physical presentation of the auditory stimulus. We thank the reviewer for raising these important points for clarification.
