# Supplementary material for: Neural dynamics differentially encode phrases and sentences during spoken language comprehension
Source: PLoS Biol. 2022 Jul 14;20(7):e3001713. doi: 10.1371/journal.pbio.3001713 (PMC9282610; doi:10.1371/journal.pbio.3001713)
Supplement: Attachment — Submitted filename: Response_to_Reviewers_2022_0420.pdf [file pbio.3001713.s004.pdf]

Reviewer #1:

SUMMARY

I thank the authors for taking the time and care to address my comments. While my concerns regarding the acoustic matching of the two primary conditions are alleviated, I still have hesitation regarding the overall claims and interpretations of the work. In their response to my review, the authors state "Our experiment tests the predictions of a computational model published in this journal (Martin & Doumas, 2017), which proposed a mechanism for building syntactic structure, time-based binding." For this to be true, I think ideally the link between the current empirical results, and the explicit predictions for the prior computational model need to be made explicit through simulation.

MAJOR

(1) Link to Martin & Doumas (2017)

This work is being used to provide empirical evidence for the claims put forward in a previous paper published in the same journal. However, to be used as such, I feel like the appropriate approach would be to take the DORA model used in the previous paper, simulate the responses to the same stimuli used in this study, and compare to the obtained EEG signals. Without a direct comparison, and without a detailed explanation of what the previously detailed "mechanism" is that is being supported here, it is very hard for the reader to validate the results.

The previous paper found that the DORA model was also able to capture the frequency tagging result of Ding et al., 2016. But to make claims regarding the binding of information through aligned responses in time, using non-isochronous stimuli, I think that we would need to see that the DORA model predicts phase alignment during the same time periods observed in the EEG responses.

Without this, I think the next best thing is to provide a schematic of the proposed mechanism, so that the reader can understand the concrete predictions without having to refer back to the previously published study.

All changes we have made to the text are highlighted therein

A (1): Thank you for encouraging us to make the predictions more accessible. We now demonstrate the predictions of time-based binding via a simulation with non-isochronous speech stimuli, and then we compare the power and phase coherence between the two conditions (please see Results on Pages 12-14 and Methods on Pages 39-40; for more discussion see Pages 52 and 64). Our simulation results support the neural data; the power and phrase coherence in the Sentence condition were

significantly higher than in the Phrase condition during an overlapping time period. We will summarize the simulations briefly here (please see the text at the aforementioned page numbers for a complete description). In response to the reviewer's concerns about Time Alignment, we will outline why finer-grained timing of phase coherence is not currently possible to estimate in any modelling framework without deeper knowledge about inter-site neural communication in the section below this one.

Fig 1a and Fig 1b show the model representation of phrases and sentences, respectively. In terms of number and type of units, sentences recruit more units than phrases do, namely in order to represent the additional constituent headed by the verb *is* in the Sentence condition. The time-based binding hypothesis predicts that phase alignment between linguistic units varies as a function of syntactic structure; differences in phase alignment arise due to a) the number of nodes needed to represent the syntactic structure and b) the relationships between nodes passing activation from 'word' to 'phrase'. From Fig 1a and Fig 1b, we see a difference in these two factors creates the binding difference, which starts directly after the input (S-units), namely with the onset of the second syllable. We calculated the phase coherence using the PO-units located in the range from 250ms to 1000 ms. The results are shown in Fig 1c through Fig 1j.

(2) Time alignment

The authors present their results time-locked to sentence onset. But the listener will distinguish sentence vs. Phrasal structure at later (and potentially multiple) points within the stimulus. In order to link the current results to a binding mechanism through phase coupling, I feel that the timing of the effects need to be directly linked to the syntactic structure available to the system at that moment in time.

A (2): We thank the reviewer for making this point. We agree that the strongest evidence for a link between model and data would be the ability to make fine-grained quantitative predictions about the magnitude of an effect and its timing. However, there are currently two major impediments to that state of affairs. First, as the reviewer is no doubt aware, the field is currently developing theories of how syntactic structure is represented and processed in the brain. This submission itself is part of that effort to gain more information about the neural encoding of syntactic structure. Thus, there is not yet a general consensus on what syntactic structure is represented, nor when it is available, nor what its neural format is. As such it is very difficult to create a continuous or discretized syntactic predictor or regressor for neuroimaging data. The same problem arises here, where it is difficult to know what syntactic information is active at a given timepoint over and above lexically-driven syntactic information. Given this uncertainty, we stick to minimal assumptions and use lexically-driven syntactic representations in our simulations and analysis – in other words, it is words being processed in DORA that in turn pass activation to phrase and sentence nodes. The difference in how activation

flows to nodes in DORA as a function of syntactic structure begins at the onset of the second syllable, or 250 ms, and persists throughout the rest of the trial/ stimulus.

The second major impediment is perhaps more difficult to address – namely it is that precise timing predictions for phase coupling are not possible to derive from any model without explicit knowledge, or at least strong assumptions, about inter-region connectivity. We do not currently have that information, so cannot exactly simulate the underlying connectivity that would give rise (or not) to the exact, fine-grained temporal pattern observed in our EEG data. *But we can simulate the boundary conditions on coupling given the representational and mechanistic assumptions made by time-based binding and DORA for representing and processing linguistic structure.*

Another way to describe this constraint is that nodes in the model represent groups of neurons that could potentially reside at different locations in the brain (viz., DORA is a model of aggregate unit activity, where nodes represent population-level responses, not the responses of individual neurons). Given the inter-site communication itself also takes time, it is possible that the phase coupling effect would vary when the two sites are located at different positions, which is currently unknowable, and may also in turn may vary slightly by individual. By conducting a simulation of the time-based binding mechanism, we could demonstrate that there is a difference at the level of phase synchronization, and can identify the beginning and end of the coupling relationships needed to represent these structures in DORA. However, without a model of inter-site connectivity on top of DORA, we cannot know exactly when and where the coupling happens in the brain. *Based on the unfolding of syntactic structure in the model alone, we show that increased power and phase coherence occurs in a time window (250-1000ms) that contains the effects observed in the EEG data (450-900ms).*

A similar timing conundrum exists for many established brain responses. For example, the P600 effect in syntactic processing is viewed as a late effect in relation to other event-related brain potentials, but we know that differences in the brain arise much earlier during syntactic processing. For example, behavioral responses during the detection of syntactic anomalies occur much earlier than the P600, and detection of syntactic violations occurs earlier than detection of semantic violations (e.g., Marslen-Wilson et al., 1988). Nonetheless, the P600 is typically observed between 600-1000 msec post-violation onset. Given this pattern, neural responses can only be seen as the upper limit on temporal processing in the brain rather than veridical one-to-one signals indicating real-time processing in the brain. Again, such a phenomenon could result from connectivity networks acting as a filter for signal detection at the scalp.

To recap, we more closely examined the phase coupling effect predicted by DORA based on time-based binding. To do this, we used non-isochronous syllables as input, then performed simulations and conducted a phase coupling analysis between PO-units with its corresponding S-units. Our comparison suggested that the phase synchronization level was significantly higher for the sentences than the phrases, the results are shown in Fig 1g and Fig 1j (for details please see Pages 39-40). The results

of the simulation demonstrate that the degree of phase synchronization between layers of nodes varies when different types of syntactic structure are processed, which was the motivation for us to conduct our exploratory study and the link between our hypothesis and experiment's results. The unpredictable temporal and spatial components in the simulation is why we needed to conduct the experiment. But we can and do show that the general principle holds, and that the period in which time-based binding/DORA shows increased coupling for sentences subsumes the time period observed in the EEG data.

We have added these discussion so that the reader can better understand the limitations on the granularity of quantitative predictions. Please see pages 13-15, 40-41, 54-55.

MINOR

- in some places I feel like the writing is unnecessarily complicated. For example, by "the acoustic and physical dynamics of speech do not injectively mark the linguistic structure and meaning that we perceive" do you mean that linguistic structure cannot be directly read out from the acoustic signal? There are a few instances where I think the language could be simplified a bit.

We have changed "injective" to "one-to-one" to communicate the meaning more accessibly.

- I found it a bit distracting the sentence "speech contains an abundance of acoustic features in both time and frequency" … speech IS an acoustic signal, which can be defined, if you choose, relative to the dimensions of time and frequency. Do you mean that it is particularly variable along these dimensions? It is not just that speech contains acoustic features, it cannot be divorced from those features.

We mean that speech can be described along many dimensions. We have changed this to "can be described by".

- Lines 1039:1042 seem circular / repetitive to me

Now lines 1324-1327; unfortunately, we do not follow what is circular here regarding the argument that alpha band modulations may reflect multiple processes as a function of context. We are happy to adapt or change the text if the reviewer could be more specific about what is circular.

- Typo "organisation" on line 1182

A: Thanks for the questions, we have revised the writing in the positions that you mentioned.

Reviewer #2:
[identifies himself as William Matchin]

I am mostly satisfied with the responses and revisions that the authors have provided to my concerns on the original submission. However, I still have some minor concerns.

In the response document the authors note that they have provided a gloss to figure 1, but I do not see this - all I see is the Dutch syllable, without information regarding the linguistic function of these elements. I feel that some of this detail should also be discussed earlier in the paper - that it's not just the hierarchical arrangement that differs between the sentence and phrase conditions, but also the addition of a function word in the sentence condition and an agreement morpheme in the phrase condition.

A: Thank you for encouraging us to be clear and accessible. We have added a high-level description of the morphemic and syntactic components through the paper, wherever we previously mentioned syntactic structure in our conditions. Please also see pages 7-9, 33-34, and 53 for changes.

In our view, in agreement with Reviewer 2, it is morphemic differences (i.e., an inflectional morpheme *-e* on the adjective *rode* in the Phrase condition and a function word/ verb/ inflectional verb phrase headed by *is* in the Sentence condition) that cue the construction or inference of hierarchy (and syntactic structure more generally). In our experiment, we were able to preserve these features while making the speech stimulus as energetically, acoustically, and spectro-temporally indistinguishable as possible. The necessity of the morphemes to form the different syntactic structures does indicate that it is likely impossible to fully orthogonalize morphemic and syntactic information in natural languages; we have added discussion of this aspect on Pages 7, 9, 33-34, and 53. In sum, we agree with the reviewer that the presence of morphemic differences, despite otherwise high physical similarity, is important to discuss. It is indeed interesting that the presence of these morphemes does not entail statistically detectable differences in the acoustic or spectro-temporal distribution of speech. Our core finding is that when two types of syntactically different structures are cued with the same number of physically-matched syllables, the brain can still separate them in various dimension of the neural response, which has not been shown in previous studies.

Some general comments on language - the manuscript is dense and often difficult to understand for researchers less familiar with the terminology and background. For example, line 122-123, "the relative timing of neural ensemble firing is taken as an informational degree of freedom". This is hard to unpack. I also had a hard time parsing the following sentence, lines 123-125. "distributed representations that fire together

closely in time, pass activation forward to receiver ensembles, such as words, are encoded together by those receiver ensembles…". It's not clear to me if there is some sort of grammatical issue here or the sentence is just hard to understand. I believe that more plain language and concrete examples would go a long way towards making these ideas more intelligible to the reader who is less familiar with these concepts, which is particularly important given the general scope of PLoS Biology.

A: Thanks for pointing out these passages. We have revised the text to unpack the intended meaning and make the claims more accessible and explicit. The new version can be seen on Lines 125-133.

It is hard for me to interpret the claims that the physical stimuli between the phrase and sentence conditions are "highly similar", since it is not clear what the relevant baseline to me is. For example, if we take random pairs of sentences in the present stimulus set, what would the physical similarity be? I think any claims of "similar pattern", "highly similar", etc., need to be evaluated against some relevant baseline. I'm not 100% sure what that baseline should be, but of course there are going to be significant correlations between two speech stimuli of similar length. Case in point, figures 1c and 1d claim to show that the temporal-spectral pattern is similar, but they definitely have noticeable differences, for example the much stronger high frequency energy in 1d at ~220-250 ms that is missing in 1c, etc. Figure 1e makes is hard to examine the true differences in conditions because the lines are thick and squished together. On this point, it seems to me that while showing significant acoustic similarity between the conditions is relevant, this does not show that there are no differences, and that these differences might contribute to the results.

I feel that this should be acknowledged a bit more clearly in the manuscript - that it's of course impossible to rule out acoustic differences contributing to these effects, but that it's unlikely that this is happening for various reasons.

A: We understand the reviewer's concerns. Given their previous points about morphology in the previous section we think that the discussion of this point dovetails nicely with the discussion of the role of morphology.

First, other than quantitative analysis of our stimuli, we have no other way to demonstrate an effect of the nature the reviewer mentions – namely that "sub-statistical" variation in the stimulus, something that cannot be reliably physically identified in the stimulus, drives brain response (with the worry that in the limit it does so more than cognitive processing in the brain).

The idea that a physical difference that is not statistically or quantitatively characterizable is being used exclusively by the brain without the brain adding information of its own is interesting and not impossible. If the brain is perceiving

morphemic differences from the stimuli, which we believe it is, despite the physical similarity of the stimuli, it seems highly likely that the brain is doing this through perceptual inference (as in other areas of perception and cognition), where the brain adds information from stored representations in a form of bias, rather than it being the stimulus that is carrying the information.

As we were able to demonstrate that the difference in neural response between Sentences and Phrases is unlikely to be driven by the physicality of the stimulus using forward modelling, we provide an analysis that shows such “added” information from the brain and not from the physical stimulus (see the forward models by condition in Fig. 8k-l). This approach makes a strong case that differences in neural response do not stem purely from the physical stimulus itself, but from the information the brain adds through inference, possibly in response to the perception of morphemes from physically similar spectrograms. We agree this point deserves discussion and we have added to clarification and discussion of morpheme-level information cueing syntactic structure on Pages 7-9, 33-34, and 53.

To address Reviewer 2’s questions regarding baseline for the similarity comparison – we used two baselines for the similarity analysis. The first baseline is simply 1, which 1 represents the case when the two signals are exactly the same at every single time point. If we pick random pairs of sentences in our set, we checked how close the two randomly selected stimuli are by comparing it to the similarity level of two stimuli that are exactly the same. However, the first baseline itself does not make statistical sense, for instance, we don’t know whether 0.95 is higher than 0.90 for an individual pair from statistical perspective over the population of items. To solve this concern, we applied a permutation approach that was used in many studies (Maris & Oostenveld, 2007, Luo and Poeppel, 2007, Cohen, 2014), which is also the basis for frequentist inference (Rand Wilcox, 2021). Specifically, as time alignment is what we want to check, we first calculate the level of similarity in time between pairs of signals that we are interested in (e.g., phrase-sentence pairs with the same semantic components). Then we randomly selected 1000 pairs of signals to extract the maximum similarity value from the 1000 similarity values. After repeating the procedure 1000 times, we would get a distribution with 1000 similarity values. We finally check the position of the real similarity values in this distribution, if the value is higher than the threshold (97.5% in our case as it was a two-sides comparison), we would say the similarity is statistically significant (please see Methods for details). A two-dimensional example would be intuitive, suppose we wanted to check the similarity between two people in a class on a feature with two dimensions (e.g., height and weight), after repeating this permutation procedure that was mentioned above, the pairs (two people) that are highly similar in the 2d feature would be pop-out.

Frequency decomposition yields a 1/f shape, which means the amplitude decays frequencies increase. The characteristics of frequency decomposition makes low frequencies weighted higher than high frequencies in the spectrum. Comparing Fig 1c and Fig 1d at ~220-250 ms, we see a high frequency difference (~ 5 to 6 kHz,

sentence > phrase), however, if the difference also occurs at low frequencies (~ 0.5 to 1.2 kHz, phrase > sentence), when doing a summation over those time bins across all frequencies, we would get a similar energy profile (as shown in Fig 1e). The energy profile comparison is often the appropriate approach to compare similarity in temporal dimension (when with optimized sampling rate). The reason for Fig 1e included high frequency wiggles is that extracting it with super down-sampled manner would degrade frequency resolution (e.g., a 100-point sequence would give a frequency resolution of 1/100 Hz, a 400-point sequence would give a frequency resolution of 1/400 Hz), which would affect the comparison in the spectral dimension as shown in Fig 1h. We want to stress that the left side of Fig 1 (c, d, e and f) shows the features of an example phrase-sentence pair, one cannot make statistical inference using this single pair. Instead, the right side of Fig 1 (g, h, i and j) shows the similarities in both time and spectral dimension over all stimuli, which could be used to make statistical inference. However, we controlled the physical and semantic features of our stimuli with a strict manner in order to say our observed effects are not likely to be driven by any sub-statistical acoustic differences.

I found a number of typos throughout the manuscript; it would probably be best to search exhaustively for such errors.

line 126 - "extended in to" -> "extended to/into"

Line 707 - synatctic -> syntactic

figure 1 f: internsity -> intensity

Figure 9 caption: volumn -> volume

line 313: acoustsics -> acoustics

Figure 4 caption: mechnism -> mechanism

Line 504: boarder -> border

A: Thank you for drawing our attention to these. We have now corrected the typos and searched the document properly for misspellings.
